# Application of latent class analysis in assessing the awareness, attitude, practice and satisfaction of paediatricians on sleep disorder management in children in Italy

**DOI:** 10.1371/journal.pone.0228377

**Published:** 2020-02-03

**Authors:** Luana Nosetti, Maria Giovanna Paglietti, Luigia Brunetti, Luigi Masini, Stefania La Grutta, Giovanna Cilluffo, Giuliana Ferrante, Marco Zaffanello, Elisabetta Verrillo, Martino Pavone, Alessandra Cristina Niespolo, Giacomo Broggi, Renato Cutrera, on behalf of the IPRS (SIMRI)Task Force on Sleep Disordered Breathing (SDB-TF)

**Affiliations:** 1 Department of Paediatrics, University of Insubria, Varese, Italy; 2 Bambino Gesù Paediatric Hospital IRCCS, Roma, Italy; 3 Tricase Hospital Lecce–Bari, Bari, Italy; 4 Azienda Ospedaliera Santobono—Pausillipon—Napoli, Naples, Italy; 5 Institute for Research and Biomedical Innovation (IRIB)- National Research Council (CNR), Palermo, Italy; 6 Department of Health Promotion Sciences Maternal and Infant Care, Internal Medicine and Medical Specialities, University of Palermo, Palermo, Italy; 7 Paediatric Clinic University of Verona, Verona, Italy; 8 Bocconi University, Milano, Italy; University of Rome Tor Vergata, ITALY

## Abstract

**Aim:**

To identify subgroups regarding paediatricians’ awareness, attitude, practice and satisfaction about management of Sleep-Disordered Breathing (SDB) in Italy using Latent Class Analysis (LCA).

**Methods:**

A cross-sectional study was conducted on a large sample of Italian paediatricians. Using a self-administered questionnaire, the study collected information on 420 Paediatric Hospital Paediatricians (PHPs) and 594 Family Care Paediatricians (FCPs). LCA was used to discover underlying response patterns, thus allowing identification of respondent groups with similar awareness, attitude, practice and satisfaction. A logistic regression model was used to investigate which independent variables influenced latent class membership. Analyses were performed using R 3.5.2 software. A p-value<0.05 was considered statistically significant.

**Results:**

Two classes were identified: Class 1 (n = 368, 36.29%) “Untrained and poorly satisfied” and Class 2 (n = 646, 63.71%) “Trained and satisfied.” Involving paediatric pneumologists or otorhinolaryngologists in clinical practice was associated with an increased probability of Class 2 membership (OR = 5.88, 95%CI [2.94–13.19]; OR = 15.95, 95% CI [10.92–23.81] respectively). Examining more than 20 children with SDB during the last month decreased the probability of Class 2 membership (OR = 0.29, 95% CI [0.14–0.61]). FCPs showed a higher probability of Class 2 membership than PHPs (OR = 4.64, 95% CI [3.31–6.55]).

**Conclusions:**

These findings suggest that the LCA approach can provide important information on how education and training could be tailored for different subgroups of paediatricians. In Italy standardized educational interventions improving paediatricians’ screening of SDB are needed in order to guarantee efficient management of children with SDB and reduce the burden of disease.

## Introduction

Sleep-Disordered Breathing (SDB) refers to a spectrum of disorders from primary snoring to upper airway resistance syndrome, obstructive alveolar hypoventilation and frank obstructive sleep apnea syndrome (OSAS). [1,2] The overall prevalence of SDB in childhood varies among the different populations, ranging from 4 to 11%; [3] of note, data from the Italian population of children reported prevalence of snoring and OSAS ranging from 1.5% to 27.6% and from 1% to 1.8%, respectively for preschoolers [4] and school-age children. [5]

Evidence suggests that accreditation-certification of sleep centres and physicians has been associated with greater timeliness and reduced health costs, leading to more standardization of health care delivery. [6] Indeed, a strong conceptual relationship between professional education and health conditions has been suggested to be a dynamic framework aimed at improving the knowledge and practice of health professionals and performance of the health system. [7] At present, even though data from previous studies are scanty, surveys investigating knowledge, attitude and practices of paediatricians in SDB have emphasized the need for more education. According to results published by Owens et al., who performed a cross-sectional survey in a large sample of community-based and academic paediatricians, despite their acknowledgment of the importance of sleep problems, many paediatricians fail to screen adequately for them, especially in older children.[8] Similarly, Balbani et al. underlined the gap between research on SDB in childhood and clinical practice in a survey involving 112 paediatricians. [9] Moreover, when interviewed about their basic knowledge and beliefs, general paediatricians and child neuropsychiatrists expressed the need for additional education and training. [10] As a matter of fact, results on primary care surveys showed that the most frequently reported barriers to implementation of appropriate screening practices include insufficient education, poor academic medical training and also a low number of patients with these disturbances seen in primary care.[11,12] Taken together, these findings highlight that a sizeable knowledge gap among paediatricians is still recognizable. In addition, evidence suggests poor satisfaction of paediatricians regarding both knowledge and practices, [9] which could be an additional factor negatively impacting quality of care of childhood SDB.

The relationship between knowledge, beliefs, attitudes, behaviour and perceptions is complex. Studies referring to the primary care setting exploring these variables in the context of SDB management used data from questionnaires [13–15] which were basically computed as the percentage of correct answers or by means of a Likert scale or using some statistical technique, such as principal component analysis, for finding patterns in large batches of data. [16] However, rather than a variable-centred approach, the use of a person-centred statistical method like Latent Class Analysis (LCA) could offer an advantage for identifying latent class membership among participants with multivariate categorical data. Indeed, LCA is a method which assigns respondents to classes based on their responses to items in the questionnaire, rather than them being arbitrarily assigned to classes by the researchers.[17] Some studies used this approach for identifying subgroups of physicians relative to their competency or satisfaction. [18,19] This kind of approach is therefore more suitable for investigating research questions and hypotheses aimed at categorizing a heterogeneous population into subpopulations based on chosen variables. In particular, our research questions were the following: what subgroups of paediatricians can be identified with regard to dimensions of awareness, attitude, practice and satisfaction of SDB? What is the relation between belonging to these subpopulations and a set of covariates? Identifying subgroups could be useful for developing intervention specifically targeting paediatricians’ needs and requirements.

No studies evaluating classes of paediatricians with regard to awareness, attitude, practice and satisfaction about SDB management in children by means of LCA have been performed so far.

Therefore, the aim of this study was to investigate latent classes of awareness, attitude, practice and satisfaction among a large sample of Italian paediatricians in order to provide basic evidence for future potential intervention strategies.

## Methods

### Study design

Between January and December 2015, the Italian Paediatric Respiratory Society, IPRS (SIMRI), created an inter-professional Task Force (TF) composed of sleep physicians, advanced practice nurses and medical assistants practicing in the field of SDB, aiming to explore: practice among health professionals; develop education; determine educational needs. Paediatric Hospital Paediatricians (PHPs) and Family Care Paediatricians (FCPs) who were IPRS (SIMRI) members took part in the SDB-TF electronic survey. The study was announced during the annual meeting of the Society held in October 2015. Participation in the study was voluntary and anonymous–no names or individually identifying information were recorded. From the beginning of 2016, interested PHPs and FCPs were asked to login to the IPRS (SIMRI) website in order to take the online survey.

### Ethics statement

The study was submitted for approval in 2015 by the principal investigator (LN) to the IPRS (SIMRI) Scientific Board. Participants gave their consent for data publication.

### Construction of the self-administered questionnaire (SAQ)

The Self-administered Online Questionnaire (SAQ) “*SleepPed questionnaire*” was constructed by the SDB-TF by adapting other existing and validated questionnaires for assessing sleep knowledge in a medical education setting. [20–22] The SAQ was constructed in four phases over a 12-month period, including initial item selection, expert board review, pilot testing for assessment of the reliability and validity, and final item selection and validation.

The SAQ was structured in four main sections: *awareness* concerning SDB (section A); *attitude* concerning the SDB diagnosis process (section B); *practice* with the SDB treatment approach (section C); assessment of *satisfaction* regarding management of SDB (section D) (see S1 Fig). The SAQ was prepared as a specific form to be filled in online (closed format questions) after registration on the IPRS (SIMRI) website (www.simri.it).

*Section* A, addressing *awareness*, was made up of questions about the relevance of the SDB problem: A1) “*Do you think SDB is a problem*?” (possible replies to the questions were “*not relevant” or “not very relevant” or “relevant” or “very relevant”);* A2) “*In your clinical practice*, *do you consider a multidisciplinary pathway in the management of a child with SDB*?*”*; A3) *“According to you*, *are the parents of children with SDB aware of potential serious complications of SDB*?*”*; A4) “*Would you be interested in attending a training course on SDB*?” Possible replies to questions A2, A3 and A4 were “*yes” or “no*.*”*

*Section* B investigated *attitude* on the SDB diagnostic process using the following questions: B1) “*How do you make an SDB diagnosis*?*”* Possible replies to the question were “*clinical evidence" and* “*instrumental examination”; “In your clinical practice*, *how often did you make a diagnosis for each of the following kinds of SDB in the last 12 months*? *(*B2) *OSAS*, B3) *snoring*, B4): *Apparent Life-Threatening Event (ALTE)*?*”* Possible replies to the questions were “*never” or “rarely” or* “*often*.*”*

*Section* C investigated SDB *practice* using the following question “*Managing patients with SDB*, *how often did you propose the following treatment in the last 12 months*?” regarding several treatment approaches, i.e. the following: C1) drugs (i.e. nasal corticosteroids), C2) adenoidectomy, C3) adenotonsillectomy, C4) weight loss and C5) non-invasive ventilation (NIV). Possible answers were “*never” or “rarely” or* “*often” or* “*very often*.*”*

*Section* D assessed *satisfaction* regarding SDB management (D1) and investigated using the following in the last 12 months: D2) night pulse oximetry (home and sleep lab); D3) polygraphy concurrently monitoring cardiorespiratory outputs (home and sleep lab); D4) complete polysomnography (PSG) with EEG (home and sleep lab). Possible answers were “*yes” or “no*.*”* See S2 Fig for details.

### Pilot study

Confirmatory factor analysis (CFA) was used to investigate the construct validity of the SAQ [23]. Model fitting was evaluated using the comparative fit index (CFI) [24], the Tucker Lewis index (TLI) [25], and the root mean square error of approximation (RMSEA) [26]. For CFI and TLI, values above 0.90 were considered as acceptable fits, and above 0.95 as good fits. RMSEA was considered as an acceptable or good fit if it was equal to or less than 0.08 [27], and equal to or less than 0.05, respectively. CFA was performed on a sample of 102 paediatricians; the characteristics of the sample are reported in Nosetti et al. 2019 [28]. Five models were developed to find the best fit. Model 1 was a one latent factor model. Model 2 was a two-factor model with awareness and satisfaction as latent factors. Model 3 was a three-factor model with awareness, attitude and satisfaction as latent factors. Model 4 was a four-factor model with awareness, attitude, practice and satisfaction as latent factors. Finally, model 5 was a five-factor model with awareness, attitude, practice, knowledge and satisfaction as latent factors. According to the fit indices (Table 1), Model 4 was the best model, since it had a lower RMSEA value (0.049), and higher CFI (0.923) and TLI values (0.903). These findings showed good model fitting, supporting the four hypothesized latent constructs of awareness, attitude, practice and satisfaction.

**Table 1:** Fit indices from CFA.

	RMSEA	90% CI	CFI	TLI	AIC	BIC
Model 1	0.089	0.067–0.111	0.721	0.675	2554.175	2632.924
Model 2	0.090	0.068–0.112	0.719	0.668	2555.827	2637.201
Model 3	0.090	0.067–0.112	0.728	0.671	2555.566	2642.190
**Model 4**	**0.049**	**0.000–0.077**	**0.923**	**0.903**	**2507.595**	**2602.094**
Model 5	0.050	0.000–0.079	0.920	0.896	2511.346	2613.720

### Statistical analysis

Data were presented as no. (%) and were depicted using radar plots. Differences of categorical variables were analyzed using the Chi-squared test.

LCA was used to discover underlying response patterns, thus allowing identification of respondent groups with similar awareness, attitude, practice and satisfaction. LCA was computed using the R poLCA package, which estimates the latent class model by maximizing, with respect to *p_r_* and *π_jrk_*, the following log-likelihood function:

$$\ln L=\sum_{i=1}^{N}\ln\sum_{r=1}^{R}p_{r}\prod_{j=1}^{J}\prod_{k=1}^{K_{j}}\pi_{jrk}Y_{ijk}$$

where J indicates the polytomous categorical variables (the “manifest” variables), each of which contains K_j_ possible outcomes, for individuals i = 1…N; *Y_ijk_* denotes the observed values of the J manifest variables such that *Y_ijk_* = **1** if respondent *i* gives the k-th response to the j-th variable, and otherwise *Y_ijk_* = **0**; *π_jrk_* denotes the class-conditional probability that an observation in class r = 1….R will produce the k-th outcome on the j-th variable and *p_r_* indicates the R mixing proportions.

The method assumes that all associations between the included variables are entirely due to the existence of distinct subpopulations called latent classes (LCs). Within the LCs all variables are assumed to be independent. [29] Based on the four aforementioned SAQ sections, four fields of interest were defined: (i) *awareness* concerning SDB (4 items from section A); (ii) *attitude* concerning the SDB diagnostic process (4 items from section B); (iii) *practice* with the SDB treatment approach (5 items from section C); (iv) assessment of *satisfaction* regarding SDB management (4 items from section D). Since “*never*” and “*very often*” were less frequently indicated by respondents, “*never*” or “*rarely*” and “*often*” or “*very often*” were aggregated. The responses to these items (i.e. manifest variables) were used to categorize respondents into groups with similar response profiles (i.e. LCs). Variable selection for LCA was performed in order to find the set of variables with relevant clustering information and discard those that were redundant and/or not informative. To perform variable selection we started from a full model deleting variables which were not statistically different between the classes in the various LCA solutions.

Determination of the number of classes depends on a combination of factors including fit indices, class size and interpretability [30]. Model selection was performed using the Bayesian Information Criterion (BIC), the Akaike Information Criterion (AIC), consistent AIC (cAIC) and entropy [31] and G^2^. According to the recommended fit indices, the optimal class solution would have the lowest BIC, AIC and cAIC values, a relatively higher entropy [32–34] and generally the lowest G^2^ [35] and conceptual and interpretive meaning. Conditional probabilities and posterior probabilities, the probability of latent class membership for each respondent, were calculated

A logistic regression model was used to investigate which independent variables influenced latent class membership (dependent variable). A full model was estimated including the following independent variables: the specialist most frequently involved (by more than 10% of paediatricians) in the multidisciplinary pathway, i.e. pneumologist (no: reference or yes), otorhinolaryngologist (no: reference or yes), allergist (no: reference or yes) and neuropsychiatrist (no: reference or yes); number of children examined with SDB in the last 12 months (<20: reference or ≥20) and being FCPs (PHPs: reference or yes). Using a stepwise procedure based on the AIC a reduced model was obtained. The odds ratios (ORs) and their relative 95% confidence intervals (CIs) were used to describe the strength of the associations. Class 1 was used as the reference group.

Analyses were performed using R 3.5.2 software. A p-value <0.05 was considered statistically significant.

## Results

### Characteristics of study population

A total of 1014 paediatricians participated in the survey: 594 FCPs (59%) and 420 PHPs (41%) (Table 2).

**Table 2:** Awareness, attitude, practice and satisfaction of Paediatric Hospital Paediatrician (PHPs) *vs*. Family Care Paediatricians (FCPs).

	All n = 1014	Paediatric Hospital Paediatrician (PHPs) n = 420	Family Care Paediatricians (FCPs) n = 594	p-value
*Awareness*				
A1) Do you think SDB is a problem that is:				**0.02324**
Not relevant	4 (0.39%)	0 (0%)	4 (0.67%)	
Little relevant	88 (8.68%)	26 (6.19%)	62 (10.44%)	
Relevant	721 (71.1%)	314 (74.76%)	407 (68.52%)	
Very relevant?	201 (19.82%)	80 (19.05%)	121 (20.37%)	
A2) In your clinical practice, do you consider a multidisciplinary pathway in the management of a child with SDB?	542 (53.45%)	167 (39.76%)	375 (63.13%)	**<0.001**
A3) According to you, are parents of children with SDB aware of potential serious complications of SDB?	320 (31.56%)	72 (17.14%)	248 (41.75%)	**<0.001**
A4) Would you be interested in attending a training course on SDB?	800 (78.8%)	382 (90.95%)	418 (70.37%)	**<0.001**
*Attitude*				
B1) How do you make an SDB diagnosis?, n (%)				*0*.*06415*
clinical evidence	444 (44%)	169 (40.24%)	275 (46.3%)	
clinical evidence and instrumental examination	570 (56%)	251 (59.76%)	319 (53.7%)	
In your clinical practice, how often did you make a diagnosis for each following SDB in the last 12 months?				
B2) OSAS				0.60787
never	51 (5%)	20 (4.76%)	31 (5.22%)	
rarely	685 (68%)	278 (66.19%)	407 (68.52%)	
often	278 (27%)	122 (29.05%)	156 (26.26%)	
B3) Snoring				**0.00735**
never	13 (1%)	5 (1.19%)	8 (1.35%)	
rarely	156 (15%)	47 (11.19%)	109 (18.35%)	
often	845 (83%)	368 (87.62%)	477 (80.3%)	
B4) ALTE				*0*.*05583*
never	328 (32%)	119 (28.33%)	209 (35.19%)	
rarely	595 (59%)	258 (61.43%)	337 (56.73%)	
often	91 (9%)	43 (10.24%)	48 (8.08%)	
*Practice*				
Managing patient with SDB, how often did you propose the following treatment in the last 12 months?				**0.00111**
C1) Drugs, n (%)				
never	97 (9.7%)	35 (8.33%)	62 (10.44%)	
rarely	369 (36.4%)	135 (32.14%)	234 (39.39%)	
often	471 (46.4%)	204 (48.57%)	267 (44.95%)	
very often	77 (7.5%)	46 (10.95%)	31 (5.22%)	
C2) Adenoidectomy, n (%)				**<0.001**
never	43 (4.3%)	10 (2.38%)	33 (5.56%)	
rarely	413 (40.7%)	144 (34.29%)	269 (45.29%)	
often	495 (48.8%)	225 (53.57%)	270 (45.45%)	
very often	63 (6.2%)	41 (9.76%)	22 (3.7%)	
C3) Adenotonsillectomy, n (%)				**0.00061**
never	52 (5.1%)	10 (2.38%)	42 (7.07%)	
rarely	572 (56.4%)	231 (55%)	341 (57.41%)	
often	359 (35.4%)	160 (38.1%)	199 (33.5%)	
very often	31 (3.1%)	19 (4.52%)	12 (2.02%)	
C4) Weight loss, n (%)				**0.00041**
never	175 (17.3%)	50 (11.9%)	125 (21.04%)	
rarely	451 (44.5%)	207 (49.29%)	244 (41.08%)	
often	351 (34.6%)	143 (34.05%)	208 (35.02%)	
very often	37 (3.6%)	20 (4.76%)	17 (2.86%)	
C5) NIV, n (%)				**<0.001**
never	667 (65.8%)	315 (75%)	352 (59.26%)	
rarely	292 (28.8%)	94 (22.38%)	198 (33.33%)	
often	48 (4.7%)	9 (2.14%)	39 (6.57%)	
very often	7 (0.70%)	2 (0.48%)	5 (0.84%)	
*Satisfaction*				
D1) Are you satisfied with the way you manage SDB patients?	597 (58.87%)	171 (40.71%)	426 (71.72%)	**<0.001**
In your clinical practice did you perform the following instrumental tests on a child in the last 12 months?				
D2) Night pulse oximetry	570 (56%)	246 (58.57%)	324 (54.55%)	0.22682
D3) Polygraphy concurrently monitoring cardiorespiratory outputs	527 (52%)	234 (55.71%)	293 (49.33%)	*0*.*05218*
D4) PSG with EEG	477 (47%)	207 (49.29%)	270 (45.45%)	0.25424

Data are presented as no. (%). SDB: Sleep Disordered Breathing, OSAS: Obstructive Sleep Apnea Syndrome, ALTE: Apparent Life-Threatening Event, PSG: polysomnography; p-value comes from a Chi-squared test, p-values in bold were statistically significant.

With regard to section A, *awareness*, more than 90% of the respondents recognized that SDB was a significant or a very relevant problem in their clinical practice, about 79% were interested in attending a training course, and 53.5% considered a multidisciplinary pathway in management of a child with SDB and collaborated with specialists (S1 Fig). Paediatricians that recognized SDB as a significant or very relevant clinical problem were more frequently interested in attending a training course than those that saw SDB as a minor or not very relevant problem in their practice (82.1% *vs*. 46.74%, p<0.001). PHPs more frequently reported that SDB was a relevant problem in their practice (74.76% *vs*. 68.52%, p = 0.02) and they were more frequently interested in attending a training course (90.95% *vs*. 70.37%, p<0.0001) than FCPs.

About section B, *attitude*, 44% of the respondents only made an SDB diagnosis according to clinical evidence, while 56% made the SDB diagnosis combining both clinical evidence and instrumental examination. Snoring was the most frequently diagnosed disorder (83%); OSAS was diagnosed by 27% of participants and ALTE by 9%. PHPs more frequently diagnosed snoring than FCPs (87.62% *vs*. 80.3%, p = 0.007). No differences were found for OSAS and ALTE diagnosis.

The results for section C, *practice*, showed that drugs were proposed by 54% of paediatricians, adenoidectomy by 55%, adenotonsillectomy by 38%, weight loss by 39% and NIV by 6%.

Lastly, with regard to section D, *satisfaction*, 59% of respondents were satisfied with the way of managing children with SDB; 56% of paediatricians used nocturnal pulse oximetry, 52% used nocturnal polygraphy concurrently monitoring cardiorespiratory outputs, and 47% performed complete PSG with EEG. FCPs were more satisfied on how the SDB was managed than PHPs (71.72% *vs*. 40.71% p<0.001). No differences were found regarding use of night pulse oximetry, polygraphy concurrently monitoring cardiorespiratory outputs and complete PSG with EEG.

Overall, respondents interested in attending a training course compared to non-interested ones proposed drugs more frequently (58% *vs*. 39.25%, p<0.001), adenoidectomy (58.25% *vs*. 42.99%, p<0.001) and PSG (50.12% *vs*. 35.51%, p<0.001); moreover, during the last 12 months, they more frequently observed OSAS (29.88% *vs*. 18.22%, p<0.001), snoring (87.38% *vs*. 68.22%, p<0.001) and ALTE (10.25% *vs*. 4.21%, p<0.001) than non-interested ones.

### Comparison of paediatricians’ awareness, attitude, practice and satisfaction

**Fig 1:**
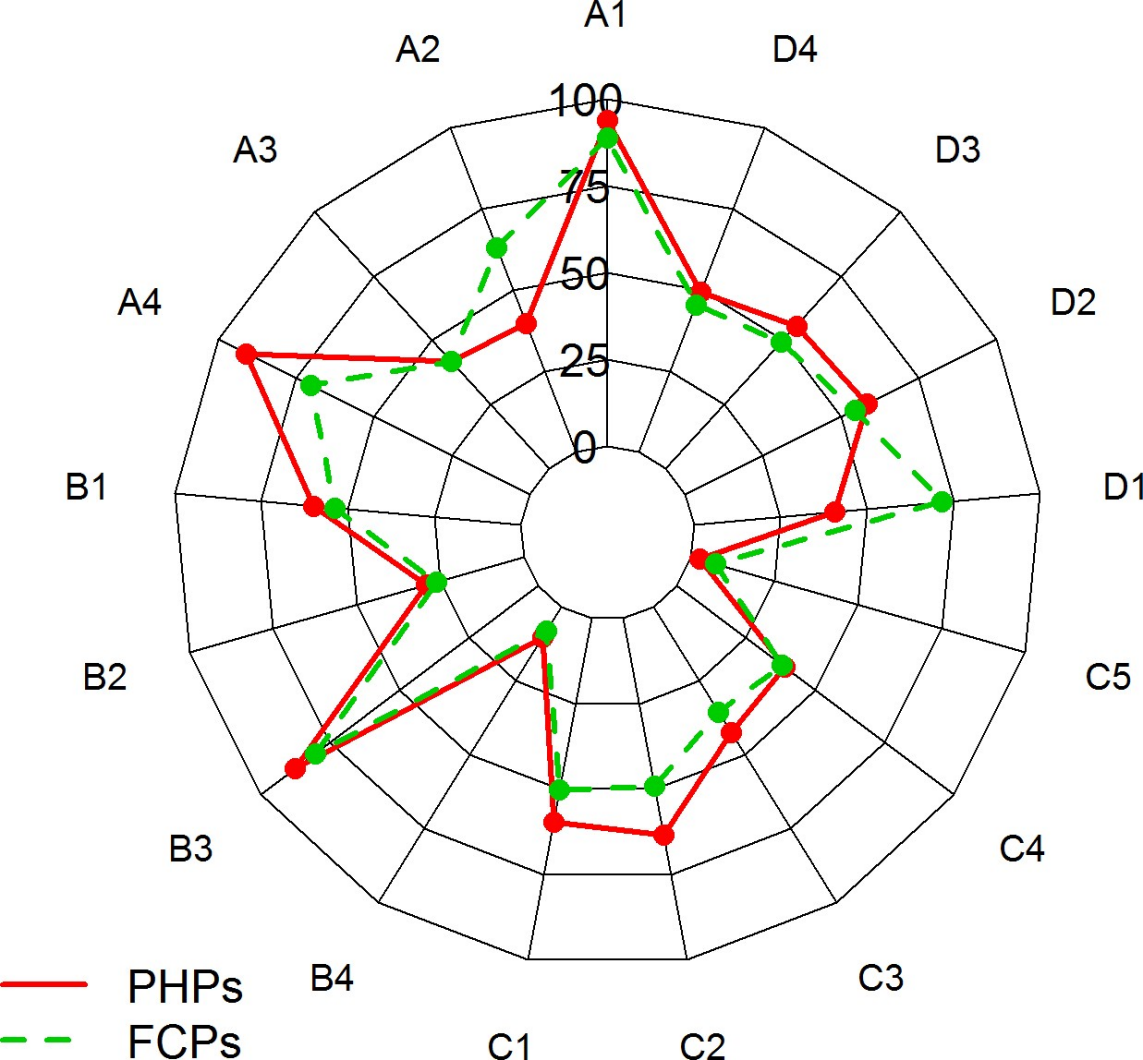
Radar plot comparing PHP and FCP answers to the SleepPed questionnaire. PHPs: Paediatric Hospital Paediatricians; FCPs: Family Care Paediatricians.

When compared to FCPs, PHPs more frequently used drugs (5.22% *vs*. 10.95%, p = 0.001), adenoidectomy (3.70% *vs*. 9.76%, p<0.001), adenotonsillectomy (2.02% *vs*. 4.25%, p = 0.0006) and weight loss (2.86%, *vs*. 4.76%, p = 0.0004). By contrast, FCPs were more inclined to suggest NIV than PHPs (6.57% *vs*. 2.14%, p<0.001) (Table 2). The answers given by PHPs and FCPs are visually reported in Fig 1.

### Model selection

The model fit statistics derived from LCA suggested that a two-class model was favoured by the lowest fit indices and high entropy value (Table 3).

**Table 3:** BIC and class size of different latent class models.

	BIC	AIC	cAIC	G^2^	Entropy	Class size
1-CLASS	12713.72	12659.58	12724.72	1978.422	-	100%
2-CLASS	12227.76	12114.56	12250.76	1409.404	0.740	64%, 36%
3-CLASS	17952.1	17706.02	18002.10	4397.942	0.722	11%, 36%, 53%
4-CLASS	17823.7	17493.95	17890.70	4151.876	0.746	11%, 35%, 35%, 19%

### Latent Class Analysis (LCA)

**Fig 2:**
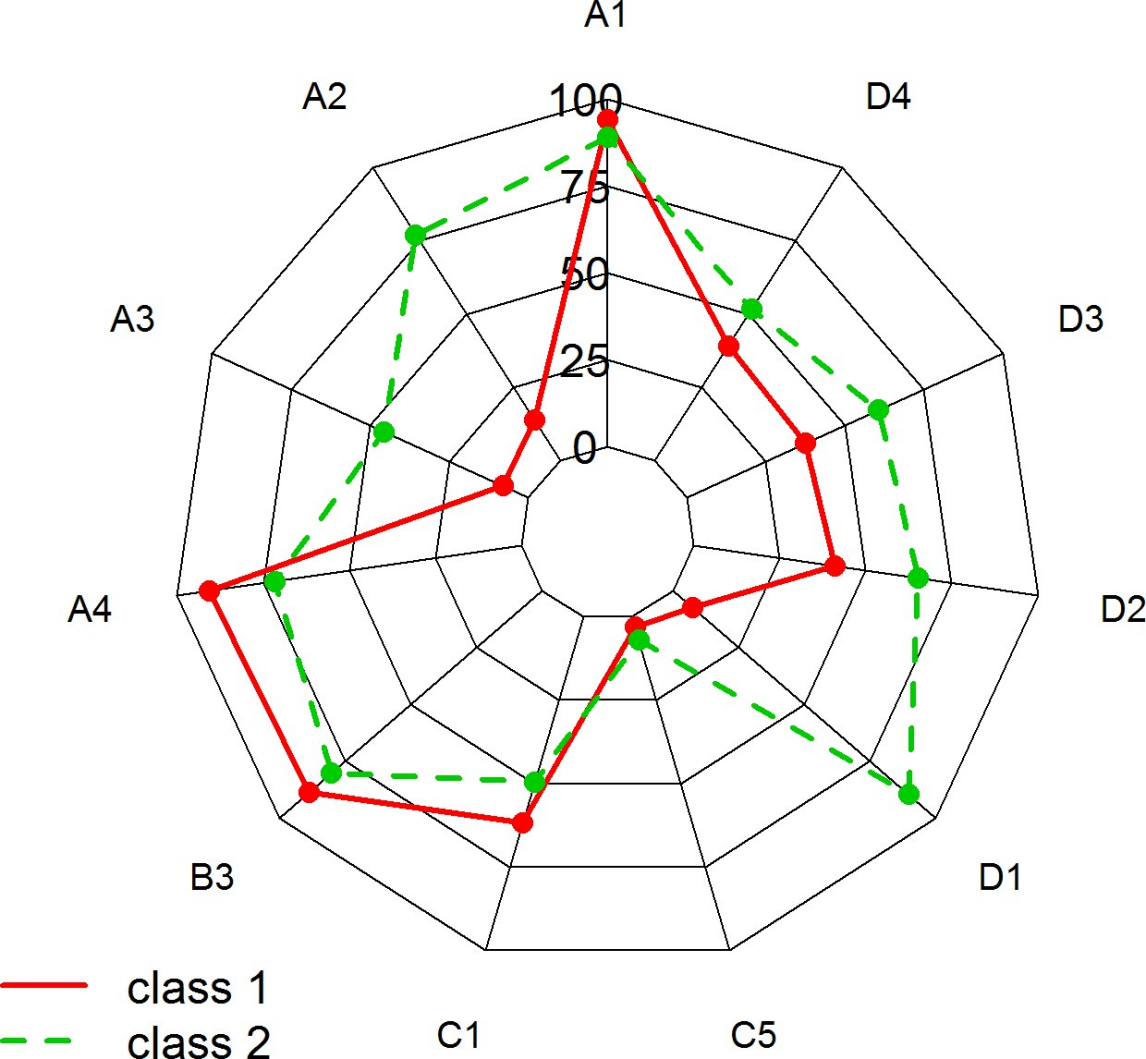
Radar plot comparing Class 1, “Untrained and poorly satisfied” and Class 2 “Trained and satisfied”.

Fig 2 illustrates the two classes identified using the LCA. To simplify the discussion, each class was assigned a summary label.

**Class 1 (no. = 368, 36.29%) “Untrained and poorly satisfied”** was composed mainly by PHPs (62.5%) and was comprised of respondents who reported a low rate of multidisciplinary approach in management of children with SDB (13.7%); they reported that parents of children with SDB were not aware of potential serious complications of SDB (7.96%); they were poorly satisfied with the way of managing SDB (7.39%); they were more interested in attending training courses (90.83%) and they did not propose night pulse oximetry (41.02%), polygraphy concurrently monitoring cardiorespiratory outputs (37.41%) and PSG (39.24%) in the last 12 months.

**Class 2 (no. = 646, 63.71%) “Trained and satisfied”** was composed mainly by FCPs (70.6%) and was characterized by respondents who reported a high rate of multidisciplinary approach (77.08%); they reported that parents of children with SDB were aware of potential serious complications of SDB (45.60%); they also reported a high level of satisfaction (89.51%) and they more frequently proposed night pulse oximetry (65.25%), polygraphy (60.63%) concurrently monitoring cardiorespiratory outputs and PSG with EEG (51.68%) in the last 12 months. The 2-class solution yielded a high class membership probability for the majority of participants (S2 Fig).

### Logistic regression

**Fig 3:**
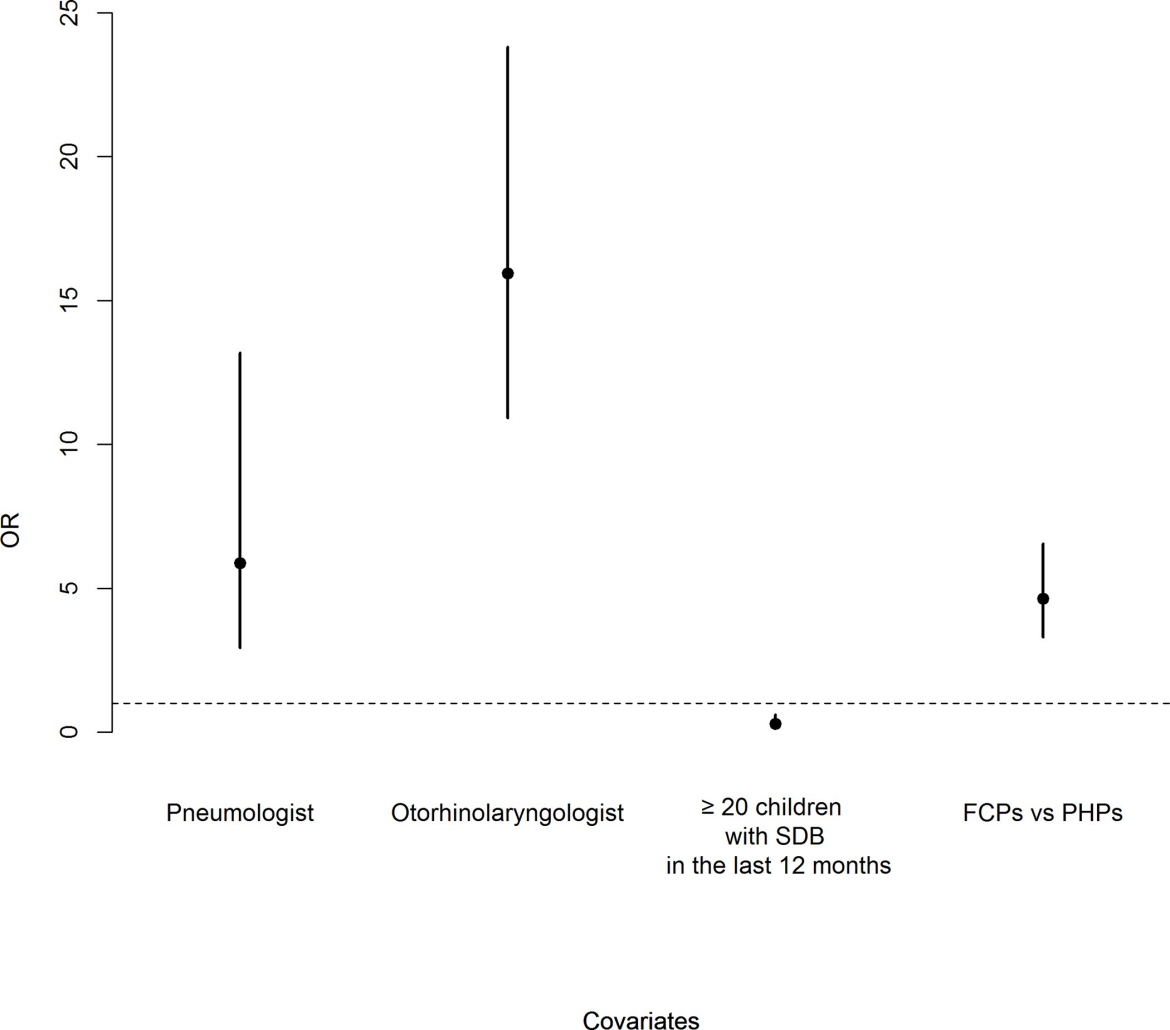
OR and 95% confidence intervals from the logistic regression for Class 2 membership. PHPs: Paediatric Hospital Paediatricians; FCPs: Family Care Paediatricians; SDB: Sleep-Disordered Breathing.

The estimated Odds ratios (ORs) and 95% CIs for class membership are reported in Fig 3. In particular, having a paediatric pneumologist as a reference professional consultant was associated with an increased probability of Class 2 membership (OR = 5.88, 95% CI [2.94–13.19]), and having an otorhinolaryngologist as a reference professional consultant was associated with an increased probability of Class 2 membership (OR = 15.95, 95% CI [10.92–23.81]). Having examined more than 20 children with SDB during the last 12 months decreased the probability of Class 2 membership (OR = 0.29, 95% CI [0.14–0.61]). FCPs showed a higher probability of Class 2 membership than PHPs (OR = 4.64, 95% CI [3.31–6.55]).

## Discussion

LCA is a statistical method for identifying unmeasured class membership among subjects using categorical and/or continuous observed variables. LCA offers several advantages over other clustering methods, allowing the comparison to be statistically tested, so that the decision to adopt a particular model is less subjective. [36] Our results obtained from LCA highlight that awareness, attitude, practice and satisfaction of the management of SDB are heterogeneous among Italian paediatricians. Specifically, we identified two LCs related to trained paediatricians with a high level of satisfaction and high rate of multidisciplinary approach, compared to untrained ones who were more interested in attending training courses and reported a low level of parent awareness about potential serious complications of SDB. Some similarities were found in *attitude*, namely in frequency of OSAS diagnosis and use of instrumental examination for SDB diagnosis such as night pulse oximetry and PSG with EEG. Conversely, significant differences between PHPs and FCPs were observed in all the questionnaire items investigating *awareness* and *practice*. Being FCPs, as well as consulting paediatric pneumologists or otorhinolaryngologists, were the factors associated with LC membership of trained and satisfied paediatricians, whereas having examined a high number of patients with SDB in the last 12 months seemed to be a factor reducing the probability of belonging to this class.

The detection of significant differences in the level of *awareness*, with a higher number of FCPs recognizing SDB as a relevant or even a very relevant problem in their clinical practice suggests that they are in a unique position for early SDB detection. Indeed, the relevance of identification of this topic was recently addressed in a large survey conducted in Italy highlighting the high priority of SDB in real practice. [37] In addition, FCPs involved in the present study were those more frequently reporting parental awareness of potential serious complications of SDB. This could be due to the peculiar relationship they have with the parents/caregivers of their patients and it could also help FCPs in developing a patient-centred model of care through discussion of this specific topic during routine scheduled examinations. [38] This finding is of particular interest, as a previous cohort study found a strong association between parent-reported SDB symptoms in early childhood and the risk of later behaviour problems, suggesting that early parental recognition of this complication early in life may allow paediatricians to reduce the burden of disease by screening for potentially reversible sleep problems. [39]

FCPs who most frequently considered a multidisciplinary pathway in the management of children of SDB were also interested in attending training courses, though to a lesser extent than PHPs (70.3% vs. 90.9%, p<0.001). This finding could be attributed to the fact that paediatricians working in hospital settings are more frequently asked to perform instrumental tests for diagnostic evaluation of SDB (mainly polygraphy concurrently monitoring cardiorespiratory outputs, 55.7% *vs*. 49.3%, p = 0.05), and jointly report a low level of satisfaction compared to FCPs (40.7% *vs*. 71.7%, p<0.001). Similarly to our findings, attending training or education programmes were also considered as an important facilitator to practice among healthcare professionals in a Canadian survey, in order to provide support for increasing evidence-based practice. [12]

As expected, SDB diagnosis was most frequently based on clinical evidence in both PHPs and FCPs, though a trend of significance could be observed when considering clinical evidence and instrumental measurements jointly, which was more frequently reported by PHPs. According to international data, the most frequently diagnosed disorders were snoring (83%) and OSAS (27%). [3] With regard to the frequency of SBD diagnosis, no significant differences were observed between PHPs and FCPs, except for snoring, more often diagnosed by PHPs than FCPs (87.6% *vs*. 80.3%, p = 0.007).

Overall, when analyzing *practice*, more than 50% of respondents prescribed drugs for SDB, though this treatment was more frequently reported by PHPs, who also proved to be more frequently involved in choosing treatment options, such as adenoidectomy and adenotonsillectomy; these results could be related to the higher level of complexity of cases that are generally referred to hospitals.

Prior reports on SDB management have generally originated from surveys involving primary care paediatricians, family practitioners and health care providers, showing that non-homogeneous practices are still found. In fact, prescription of pharmacological treatment for sleep disorders in childhood ranged from 81% of outpatient visits for sleep difficulties [40] to around 1% for a more comprehensive scenario of sleep disorders. [41,42] Overall, our study showed that FCPs were more prone to nonprescription therapies, such as weight loss, as well as to long-term use of NIV, suggesting that a different pattern of paediatric practice is recognizable. A dichotomous pattern of prescriptions has already been observed in previous research on the treatment of paediatric insomnia, [43] providing evidence that this issue might the target of further investigations. Recognition and treatment of SDB is crucial for the health and wellbeing of children, mainly when SDB is a comorbidity [44,45].

To our knowledge, no previous studies have identified classes of paediatricians’ awareness, attitude, practice and satisfaction. The aim of the present study was to identify subgroups of paediatricians based on their answers to the SleepPed questionnaire. The latent clustering numbers were not preset before analysis but the 2-class solution was chosen as the best trade-off between BIC, class size and interpretability. Paediatricians in Class 1, the “Untrained and poorly satisfied” group, had a poor level of performance in management of SDB. The majority were represented by PHPs (62.5%), who lacked awareness of the importance of a multidisciplinary diagnostic pathway and willingness to discuss the topic in order to solicit parental concerns. Physicians in this LC did not propose the use of instrumental diagnostic examination and reported they were poorly satisfied with the way of managing SDB, asking for more training. Therefore, systems of education should especially meet the demand of increased training for this group, providing detailed training plans through standardized educational programmes. In addition, the accreditation-certification status of sleep centres and physicians is also associated with greater timeliness and saving health costs, which leads to more standardization of health care delivery. [6]

Paediatricians in Class 2, the “Trained and satisfied” group, had a high level of performance in management of SDB. The LC was composed mainly by FCPs (70.6%) and was characterized by respondents who were equipped with good awareness of the importance of a multidisciplinary diagnostic pathway as well as being willing to discuss the topic in order to solicit parental concerns. Physicians in this LC proposed instrumental diagnostic examination and reported they were satisfied with their management of SDB. It is reasonable to suppose that in this group good coordination with specialists and the ability to build strong relationships with parents allow increased job satisfaction as well as quality of care.

The current study aimed to investigate which independent variables influenced LC membership using a logistic regression model. Being FCPs and having paediatric pneumologists or otorhinolaryngologists as reference professional consultants was associated with an increased probability of membership to LC of trained and satisfied paediatricians. Interestingly, examining more than 20 children with SDB in the last 12 months was a factor which reduced the probability of Class 2 membership, highlighting the need for additional education in the diagnostic workout aimed at integrating clinical and instrumental examination in an effective care pathway. In our opinion, the number of children with SDB visited in the last 12 months does not imply that the paediatricians are trained and therefore become aware of SDB burden and management. Similarly, this also does not imply that paediatricians are satisfied, given that we assumed that satisfaction is not linked to the amount of children visited in the last year but rather depends on availability of instrumental measurements (see Section D). Therefore, it would be reasonable to suppose that paediatricians’ work performance in SDB is not only dependent on the number of children with SDB they clinically recognize within their daily practice, but might be strongly associated with competencies achievable with specific training programmes. This phenomenon may be ascribed to poor knowledge of other competency elements which belong to standardized systems for training and evaluation of physicians. However, before implementing such systems of education, an estimation of the actual level of paediatricians’ competency in SDB is advisable.

There are several limitations to our study. Firstly, the cross-sectional design cannot verify direction of causality about the observed associations. Secondly, the outcomes of interest were self-reported, which is subject to recall bias through a questionnaire, so that future studies should use standardized and validated tools. Thirdly, although data were obtained throughout a large sample of Italian paediatricians, our findings are not representative of the entire population of paediatricians; moreover, some differences depending on various hospital settings (i.e. university hospital centres or remote hospital centres) should be addressed in future research. In addition, a possible selection bias is associated with subject recruitment, since only physicians who had an affiliation with IPRS (SIMRI) were surveyed.

However, this study benefited from an advanced statistical analysis. The use of LCA to investigate physicians’ awareness, attitude, practice and satisfaction can be considered a novel approach useful in developing the study framework. The study also used a logistic regression model in order to investigate which independent variables influenced LC membership. An additional strength is that we previously demonstrated the construct validity of the SAQ, which showed good model fitting supporting the four hypothesized latent constructs of awareness, attitude, practice and satisfaction.

Since the current study is observational and no expectations on response patterns are indicated, cross-validation was not carried out. Indeed, cross-validation only makes sense when the researcher has considerable background in the substantive area in question and if there is a finite number of candidate models. [46,47]

## Conclusion

On the basis of awareness, attitude, practice and satisfaction regarding SDB in children, paediatricians can be divided into two subgroups. The LCA approach can provide important information on how education and training could be adapted to or fitted for different paediatrician subgroups. Consistently with prior studies, Italian paediatricians are seen to be aware of the problem of childhood SDB; nonetheless, they express the need to improve their education. In future, training of paediatricians should use more specific measures according to the different level of competency in order to provide tailored training courses. At the same time, understanding work-related factors affecting paediatricians’ satisfaction may help in identifying potential intervention strategies. We provided the first evidence to show that some factors, including a multidisciplinary approach, may influence paediatricians’ skills and satisfaction within the management of SDB in children. These findings highlight the need to plan standardized educational interventions improving paediatricians’ screening of SDB in order to guarantee efficient management of children with SDB and to reduce the burden of disease.

## Supporting information

S1 Fig Word-cloud of specialists involved in the multidisciplinary pathway. (DOC)

S2 Fig Class membership probability for all participants for the 2-class model. Results demonstrate that for each of the 2 latent classes, the probability of assignment to that latent class was >0.90 on average for each participant (red points).
(DOC)

S1 File (DOC)

## Abbreviations

FCPs: Family Care Paediatricians
LCs: Latent Classes
LCA: Latent Class Analysis
PHPs: Paediatric Hospital Paediatricians
SDB: Sleep-Disordered Breathing
