# Supplementary figures and images for: Application of latent class analysis in assessing the awareness, attitude, practice and satisfaction of paediatricians on sleep disorder management in children in Italy
Source: PLoS One. 2020 Feb 3;15(2):e0228377. doi: 10.1371/journal.pone.0228377 (PMC6996829; doi:10.1371/journal.pone.0228377)

**S1**
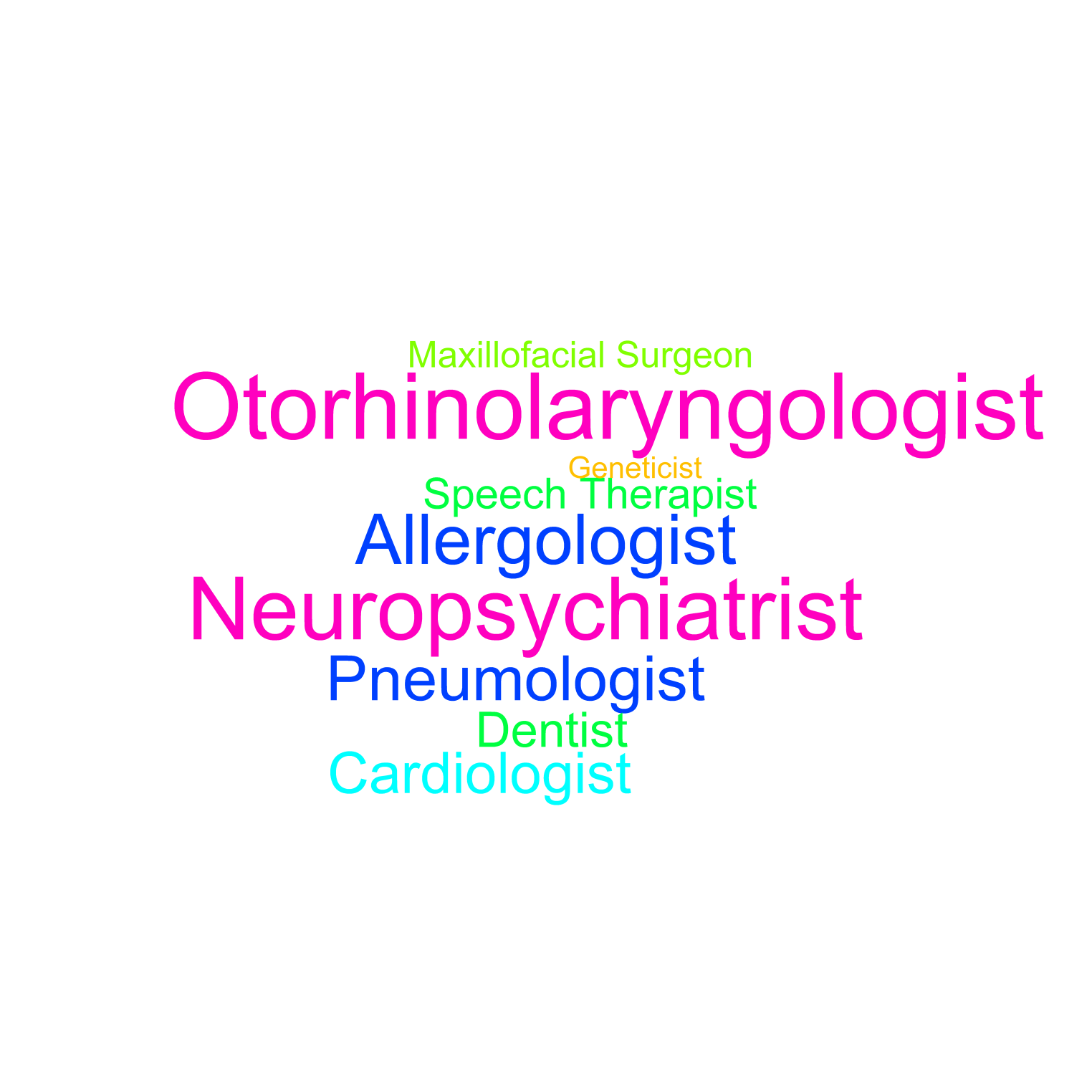
 **Fig Word-cloud of specialists involved in the multidisciplinary pathway.**

Supplement: S1 Fig — (DOC) [file pone.0228377.s001.doc]
