# Supplementary material for: Application of latent class analysis in assessing the awareness, attitude, practice and satisfaction of paediatricians on sleep disorder management in children in Italy
Source: PLoS One. 2020 Feb 3;15(2):e0228377. doi: 10.1371/journal.pone.0228377 (PMC6996829; doi:10.1371/journal.pone.0228377)

**S2 Fig Class membership probability for all participants for the 2-class model. Results demonstrate that for each of the 2 latent classes, the probability of assignment to that latent class was >0.90 on average for each participant (red points).**


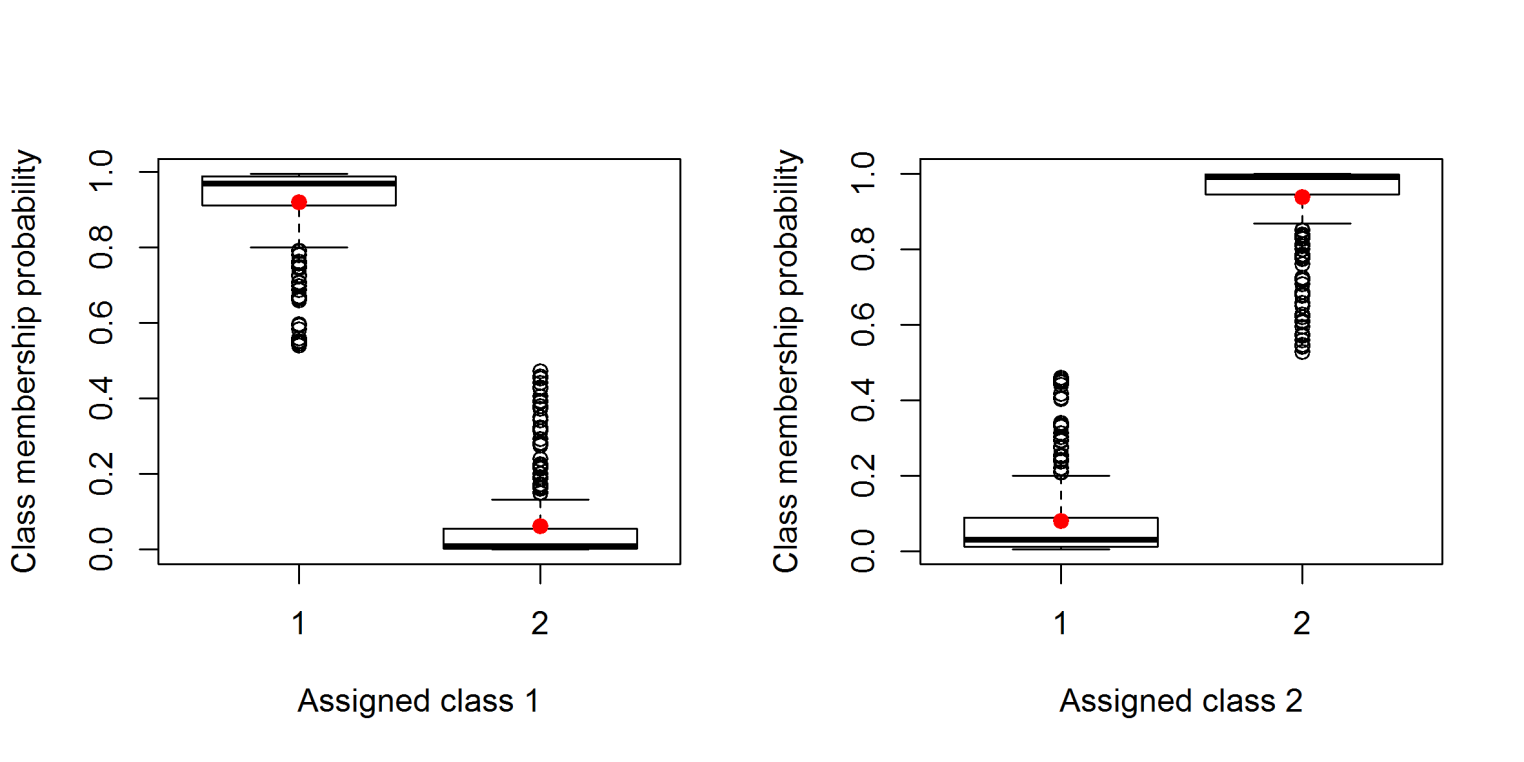

Supplement: S2 Fig — Results demonstrate that for each of the 2 latent classes, the probability of assignment to that latent class was >0.90 on average for each participant (red points). (DOC) [file pone.0228377.s002.doc]
