# Supplementary material for: Application of latent class analysis in assessing the awareness, attitude, practice and satisfaction of paediatricians on sleep disorder management in children in Italy
Source: PLoS One. 2020 Feb 3;15(2):e0228377. doi: 10.1371/journal.pone.0228377 (PMC6996829; doi:10.1371/journal.pone.0228377)
Supplement: S1 File — (DOC) [file pone.0228377.s003.doc]

***SleepPed questionnaire***

*Self-administered questionnaire (SAQ) for Family Care Pediatricians (FCPs) and Pediatric Hospital Pediatricians (PHPs) created by the SIMRI (Italian Pediatric Respiratory Society)* Task Force *on SDB (SDB-TF).*

*What is your practice status?*

- Family Care Pediatrician
- Pediatric Hospital Pediatrician

H*ow many children with sleep breathing disorders have you examined during the last 12 months?*

- <20 children
- ≥ 20 children

**Section A_Awareness**

A1) *Do you think SDB is a problem that is:*

- not relevant
- not very relevant
- relevant
- very relevant

A2) *In your clinical practice, do you consider a multidisciplinary pathway in the management of a child with SDB?*  Yes  No

*if yes, with which specialists?*

- Otorhinolaryngologist
- Dentist
- Neuropsychiatrist
- Maxillofacial Surgeon
- Pneumologist
- Geneticist
- Speech Therapist
- Allergologist
- Cardiologist

A3) *According to you, are parents of children with SDB aware of potential serious complications of SDB?*
  Yes  No

A4) *Would you be interested in attending a training course on SDB?*  Yes  No

**Section B_Attitude**

B1) *How do you make an SDB diagnosis?*

- clinical evidence
- clinical evidence and instrumental measurements

*In your clinical practice, how often did you make a diagnosis for each of the following forms of SDB in the last 12 months?*

B2) OSAS

- never
- rarely
- often

B3) Snoring

- never
- rarely
- often

B4) ALTE

- never
- rarely
- often

**Section C_Practice**

*Managing patient with SDB, how often did you propose the following treatment in the last 12 months?*

C1) Drugs:

- never
- rarely
- often
- very often

C2) Adenoidectomy

- never
- rarely
- often
- very often

C3) Adenotonsillectomy

- never
- rarely
- often
- very often

C4) Weight loss

- never
- rarely
- often
- very often

C5) Non-invasive ventilation

- never
- rarely
- often
- very often

**Section D_Satisfaction**

D1) *Are you satisfied with the way you manage SDB patients?*

- yes
- no

D2) *Did you perform the night pulse oximetry tests on a child in the last 12 months?*

- yes
- no

D3) *Did you perform poligraphy concurrently monitoring cardiorespiratory outputs on a child in the last 12 months?*

- yes
- no

D4) *Did you perform the complete polysomnography with EEG tests on a child in the last 12 months?*

- yes
- no
